# Effectiveness of fluoride sealant in the prevention of carious lesions around orthodontic brackets: an OCT evaluation

**DOI:** 10.1590/2177-6709.20.6.037-042.oar

**Published:** 2015

**Authors:** Matheus Melo Pithon, Mariana de Jesus Santos, Camilla Andrade de Souza, Jorge César Borges Leão, Ana Karla Souza Braz, Renato Evangelista de Araujo, Orlando Motohiro Tanaka, Dauro Douglas Oliveira

**Affiliations:** 1 Professor of Orthodontics, Universidade Estadual do Sudoeste da Bahia (UESB), School of Dentistry, Department of Health, Jequié, Bahia, Brazil; 2 Student, Universidade Estadual do Sudoeste da Bahia (UESB), School of Dentistry, Department of Health I, Jequié, Bahia, Brazil; 3 Postgraduate Student, Pontifícia Universidade Católica do Paraná (PUC-PR), Graduate Dentistry Program in Orthodontics, School of Health and Biosciences, Curitiba, Paraná, Brazil; 4 Postgraduate Student, Universidade Federal de Pernambuco (UFPE), Postgraduate Program in Biomedical Engineering, Recife, Pernambuco, Brazil; 5 Adjunct Professor, Universidade Federal de Pernambuco (UFPE), Postgraduate Program in Biomedical Engineering, Recife, Pernambuco, Brazil; 6 Professor, Pontifícia Universidade Católica do Paraná (PUC-PR), Graduate Dentistry Program in Orthodontics, School of Health and Biosciences, Curitiba, Paraná, Brazil; 7 Program director, Pontifícia Universidade Católica de Minas Gerais (PUC-MG), Orthodontic Graduate Program, Belo Horizonte, Minas Gerais, Brazil

**Keywords:** Dental caries, Fluorides, Pit and fissure sealants

## Abstract

**Objective:**

This article aimed to evaluate *in vitro* the efficiency of Pro Seal fluoride sealant application in the prevention of white spot lesions around orthodontic brackets.

**Material and Methods:**

Brackets were bonded to the buccal surface of bovine incisors, and five groups were formed (n = 15) according to the exposure of teeth to oral hygiene substances and the application of enamel sealant: G1 (control), only brushing was performed with 1.450 ppm fluoride; G2 (control) brushing associated with the use of mouthwash with 225 ppm fluoride; G3, only Pro Seal sealant application was performed with 1.000 ppm fluoride; G4 Pro Seal associated with brushing; G5 Pro Seal associated with brushing and mouthwash. Experimental groups alternated between pH cycling and the procedures described. All specimens were kept at a temperature of 37 °C throughout the entire experiment. Both brushing and immersion in solutions were performed within a time interval of one minute, followed by washing in deionized water three times a day for 28 days. Afterwards, an evaluation by Optical Coherence Tomography (OCT) of the spectral type was performed. In each group, a scanning exam of the white spot lesion area (around the sites where brackets were bonded) and depth measurement of carious lesions were performed. Analysis of variance (ANOVA) was applied to determine whether there were significant differences among groups. For post hoc analysis, Tukey test was used.

**Results:**

There was statistically significant difference between groups 1 and 2 (p = 0.003), 1 and 3 (p = 0.008), 1 and 4 (p = 0.000) and 1 and 5 (p = 0.000). The group in which only brushing was performed (Group 1) showed deeper enamel lesion.

**Conclusion:**

Pro Seal sealant alone or combined with brushing and/or brushing and the use of a mouthwash with fluoride was more effective in protecting enamel, in comparison to brushing alone.

## Resumo

**Objetivo:**

avaliar *in vitro* a eficiência da aplicação de selante fluoretado Pro Seal na prevenção de lesões de mancha branca ao redor de braquetes ortodônticos.

**Métodos:**

braquetes foram fixados na face vestibular de incisivos bovinos, formando os seguintes cinco grupos (n = 15) de acordo com a exposição a substâncias de higiene bucal e a aplicação do selante de esmalte: 1) controle - realizada apenas escovação; 2) controle - escovação + utilização de enxaguante bucal com flúor; 3) apenas aplicação de selante Pro Seal (Reliance Orthodontic Products, Itasca, Ill); 4) Pro Seal + escovação; 5) Pro Seal + escovação + bochecho. Os grupos experimentais alternaram entre ciclagem de pH e os procedimentos descritos. Todos os espécimes foram mantidos, durante todo o experimento, à temperatura de 37°C. Tanto a escovação quanto a imersão nas soluções foram realizadas em um intervalo de tempo de 1 minuto, seguidas por lavagem em água deionizada, três vezes por dia, durante 28 dias. Após isso, realizou-se avaliação utilizando Tomografia por Coerência Óptica (OCT) do tipo espectral (Ganymede OCT/Thorlabs, Newton, EUA). Em cada grupo, foi realizado um exame em varredura da área de mancha branca (em torno da região onde foram colados os braquetes), assim como a aferição da profundidade das lesões de cárie. A análise de variância (ANOVA) foi aplicada para determinar se houve diferenças significativas entre os grupos. Para o teste*post hoc*, foi utilizado o teste de Tukey.

**Resultados:**

houve diferença estatisticamente significativa entre os grupos 1 e 2 (p = 0,003), 1 e 3 (p = 0,008), 1 e 4 (p = 0,000) e 1 e 5 (p = 0,000). O grupo onde apenas escovação foi feita (Grupo 1) mostrou lesão do esmalte mais profunda.

**Conclusão:**

o selante ProSeal sozinho ou combinado com escovação e/ou escovação e uso de bochechos com flúor foi eficaz em proteger mais do que apenas escovar esmalte.

## INTRODUCTION

The literature has shown irrefutable evidence of the multifactorial nature of caries disease, emphasizing the combination of biological, environmental and behavioral factors determinant for its appearance.^1^ The first clinical sign of the disease is white spot lesion which results from surface mineral loss from tooth enamel.

The opaque white appearance of carious lesion on enamel can be attributed to subsurface demineralization associated to increased porosity and consequent changes in the optical properties of tooth enamel. This type of lesion occurs as a result of repeated episodes of mineral loss from the surface caused by dental biofilm and saliva, and mineral loss from the subsurface in reconstitution of enamel surface.^2^

This dynamics is not continuous, as it is interrupted by the process of remineralization in the oral cavity. Provided that enamel surface is intact, there is a possibility of reverting, or even eliminating, white spot lesion. This may occur spontaneously by means of the combined action of salivary minerals and fluoride present in dentifrices, or may be achieved by therapeutic intervention.^2^^,^ 3

Tooth enamel demineralization adjacent to orthodontic brackets is a significant clinical problem, since the presence of appliances hampers cleaning and maintenance of a healthy oral environment, as they potentiate biofilm accumulation on tooth surfaces and the gingival margin.^2^^,^ 4 ^-^ 7

These decalcified lesions result not only in unfavorable esthetics, but may also require additional restorative treatment in more severe cases.^8^

In order to diminish the risk of demineralization during orthodontic therapy, various methods are employed, in which fluoride is the main agent used in different compositions. Various studies have evaluated different types of material that release fluoride ions into the oral cavity without requiring patient's cooperation. These materials may be dentifrices, adhesives, varnishes, gels or sealants.^2^^,^ 4 ^,^ 9 ^,^ 10

According to Chang, Walsh and Freer,^2^ a layer of sealant may be used to coat the tooth surface around brackets, sealing the susceptible areas of enamel after bracket bonding, resulting in greater protection of the tooth surface.

In order to prove this fact, the objective of the present study was to evaluate the efficiency of Pro Seal fluoride sealant in the prevention of white spot lesions around orthodontic brackets when submitted to pH cycling.

## MATERIAL AND METHODS

In this *in vitro* study, 75 bovine incisors were used. They were stored in 10% formaldehyde solution for 15 days under refrigeration at 5 °C. After removing the remaining periodontal ligament, test specimens were fabricated by positioning the teeth in PVC matrices (Amanco, São Paulo, SP, Brazil), so as to enable a larger area of the buccal surface to be exposed. They were secured by self-curing acrylic resin (Jet, Artigos Odontológicos Clássico Ltda, São Paulo, SP, Brazil). After resin curing, each test specimen containing 15 bovine teeth (n = 15) was subject to abrasion with silicon carbide abrasive paper (grain 600 M, 3M, Rio de Janeiro, RJ, Brazil), under water lubrication, so that the buccal surface of interest was exposed by removing of excess resin and flattening of the enamel. With a view to maintaining standardization, the abrasion process was performed simultaneously by applying a constant force of 500 g.

After manufacturing the test specimens, enamel prophylaxis was performed with a mixture of pumice stone and water, using a rubber cup (KG Sorensen, Rio de Janeiro, RJ, Brazil) at low speed for 15 seconds. Subsequently, the samples were washed with deionized water for 15 seconds and then dried with oil-free compressed air for 15 seconds.

Next, tooth enamel surface was divided into two parts equal in area, one of which was protected with a coat of nail varnish (Risqué, São Paulo, SP, Brazil), keeping it isolated from the experimental solutions; whereas the other was used for bonding 75 orthodontic brackets. Red nail varnish was used to make it easy to identify the surface to be maintained intact. For bonding of orthodontic brackets, light-curing composite resin (Transbond XT, 3M Unitek, Monrovia, Ca, USA) was used.

**Figure 1:**
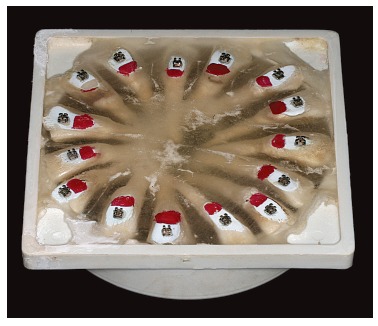
- Test specimen used in this study.

After bonding (Fig 1), the substance to be tested was applied around brackets. A sealant was used: Pro Seal (Reliance Orthodontic Products, Itasca, Ill, USA) with 1.000 ppm fluoride.

The samples were divided into 5 groups (n = 15): G1 - control, in which only brushing was performed (Colgate Total 12, São Paulo, SP, Brazil) with 1450 ppm fluoride; G2 - control, brushing and mouthwash (Plax, Colgate, São Paulo, SP, Brazil) with 225 ppm fluoride; G3 - Pro Seal sealant ; G4 - Pro Seal sealant associated with brushing; G5 - Pro Seal sealant associated with brushing and mouthwash.

All specimens of groups 1, 2, 4 and 5 were submitted to brushing with fluoride dentifrice (Colgate Total 12, São Paulo, SP, Brazil) with 1450 ppm fluoride and pH cycling. The mouthwash used was Colgate Plax Classic, with 225 ppm fluoride (Colgate-Palmolive Ind. e Com. Ltda, São Paulo, SP, Brazil). This procedure was interspersed with washing of samples with water. Brushing and immersion were performed for one minute, followed by washing in deionized water three times a day for a period of 28 days.

### pH cycling protocol

The pH cycling protocol consisted of using artificial neutral remineralizing saliva (calcium 1.54 mmol / L; phosphate 1.54 mmol / L; acetic acid 20 mmol / L, and 0.308 g of ammonium acetate, pH adjusted to 7.0 with potassium hydroxide (Vetec, Rio de Janeiro, RJ, Brazil), and demineralizing saliva (3 mmol / L of calcium; 3 mmol /L of phosphate; 50 mL acetic acid / L; ammonium acetate and 0.308 g with pH adjusted to 4.5 with sodium hydroxide (Vetec, Rio de Janeiro, RJ, Brazil).

To induce a strong cariogenic challenge, test specimens were stored in demineralizing saliva for 22 consecutive hours. After being washed with deionized water, they were kept in contact with remineralizing saliva for two hours in order to complete the 24-hour cycle. During the pH cycling period, the specimens were kept in an incubator (Fanem Ltda, São Paulo, SP, Brazil), at a constant temperature of 37 °C to simulate the oral environment. This dynamics was reproduced for a period of 28 days, during which the artificial saliva (neutral and acid) was changed every two days.^11^

### Evaluation by Optical Coherence Tomography (OCT)

The enamel microstructure was evaluated by means of Optical Coherence Tomography (OCT) using a commercial system of the spectral type (Ganymede OCT/Thorlabs, Newton, USA). The system is based on the Michelson interferometer. It is connected to a pre-configured computer and the images are obtained by means of scanning. The base unit contains a light source, which, in this case, is a Superluminescent Diode (SLD) with wavelength centered at 930 nm and spectral width of 100 nm. Using an A-scan rate of 29 kHz, this system is able to produce 29 frames per second (fps) with 512 lines per frame and an axial resolution of 55 µm. Thus, volumetric images (3D images) in transverse cuts (2D images) were produced from a scanning exam of the white spot area located around brackets.^12^

Afterwards, three linear measurements were made in different regions of each sample, corresponding to the two regions of greater depth of carious lesions identified during scanning. Then, the arithmetical mean of the three measurements was calculated. This mean was the representative value for carious lesions depth for each sample.

### Statistical analysis

Data were statistically analyzed. For each parameter evaluated, descriptive statistics was used, including mean and standard deviation. Analysis of variance with two fixed factors (two-way ANOVA) was used to assess the effect of factor group. Residuals were checked for normality by Shapiro-Wilk test, and variables with asymmetric distribution were transformed into logarithmic scale (log10). When the F-test was significant (*p* ≤ 0.05) for the group factor, we proceeded with post-hoc Tukey or Tamhane test to determine differences between groups. Tamhane test was used when data showed heteroscedasticity (*p* ≤ 0.05 for Levene's test), while Tukey test was used for cases of homocedasticity (*p* > 0.05 for Levene's test).

For all analyses, significance level was set at 5% (a = 0.05). Data were analyzed in IBM SPSS Statistics for Windows software (SPSS 21.0, 2012, Armonk, NY:. IBM Corp.).

## RESULTS

Results showed statistical differences between Group 1 and Groups 2 (*p* = 0.003), 3 (*p* = 0.008), 4 (*p* = 0.000) and 5 (*p* = 0.000). The groups in which enamel was protected by Pro Seal sealant presented the lowest carious lesion depths when compared to other groups. 

## DISCUSSION

One of the most common negative effects of orthodontic treatment with fixed appliances is the development of incipient carious lesions around brackets and orthodontic bands, especially in patients with impaired oral hygiene. White spot lesions are characterized by opacity and mineral loss when compared to healthy enamel.^4^ In an attempt to minimize or even avoid the appearance of those lesions, the industry has developed products in the form of sealants. Although they are widely used by orthodontists, there is little scientific proof of the effectiveness of these materials. Based on this premise, the present study was proposed.

In this research, an evaluation was performed with OCT, a method that provides high resolution and high-definition images, in addition to being fast and noninvasive and providing an in-depth and detailed analysis of enamel microstructure. At present, it is widely used in Medicine; however, there have been a few studies conducted in Dentistry.^12^

Previous studies reveal that white spot lesions may develop within only one month,^13^^,^ 14 and there is divergence among authors about the incidence of this initial carious lesions in patients undergoing orthodontic treatment.^15^^,^ 16 ^,^ 17

For this reason, clinicians have used various products as preventive measures, in an endeavor to reduce the appearance of white spot lesions.^14^^,^ 15 Some have used topical fluoride for prevention of initial carious lesions and in enamel remineralization, and have achieved good results;^13^ however, when patient's cooperation is a factor to consider, there is a drop in the success rate of this type of treatment.^18^ Another product widely used is fluoride varnish. The application of fluoride varnishes must follow a routine, although these varnishes have shown not to be able to prevent the occurrence of white spot lesions in all cases.^19^

Therefore, as the application of sealants prevents caries formation in pits and fissures, resin sealants have been used on smooth tooth surfaces around orthodontic brackets to reduce enamel demineralization. Previous editions of resin sealants had a low performance as regards resistance to wear; however, new material has been released on the market with promises of changing this premise.^4^

One example of this new generation of products is Pro Seal light-curing fluoride sealant which is more resistant to abrasion due to having better consistence. In the comparative study by Hu and Featherstone,^20^the result found was that the degree of demineralization of enamel coated with Pro Seal was significantly lower than that found in fluoride varnish and control sealant groups.

The results found in the present study (Table 1) showed that the depths of white spot lesions developed around orthodontic devices during the pH cycling process in the group with sealant application was slightly lower when compared to those found in the brushing group. The results of the present study corroborate those of the previously mentioned study.^20^ Group 2, which was treated with brushing and mouthwash, did not differ statistically from groups that received Pro Seal sealant alone or a combination of brushing and mouthwash.

**Table 1:** - Test specimens used in this study.

**Groups**	**Treatment**	**Mean (SD)**	Statistics*
1	B = brushing	402.82 (78.19)	-2/*p*= 0.003* -3/*p*= 0.008* -4/*p*= 0.000* -5/*p*= 0.000*
2	B + MW = brushing associated with mouthwash	327.20 (50.67)	-3/*p*= 0.999 -4/*p*= 0.287 -5/*p*= 0.223
3	FS = fluoride sealant	332.95 (44.29)	-4/*p*= 0.170 -5/*p*= 0.126
4	FS + B = fluoride sealant + brushing	287.08 (53.91)	-5/*p*= 1.000
5	FS + B + MW = fluoride sealant + brushing + mouthwash	284.19 (43.32)	

S.D. = Standard deviation. *= statistically significant differences (*p* < 0.05).

In a laboratory environment, it was found that Pro Seal has the capacity of withstanding the changes in pH and be resistant to abrasion produced by the tooth brush.^20^ This result corroborates the present study, since the groups in which Pro Seal sealant was applied presented the lowest carious lesion depth values. In another study, it was found that Pro Seal has a greater capacity to protect enamel against the demineralization process than fluoride varnish or resin sealant of a fluid consistence, reducing the depth of carious lesion up to 92% in comparison to controls.^21^

In the study conducted by Knosel et al,^22^ Pro Seal was not more effective when compared to Maximum Cure^TM^ chemically activated sealant which proved to be more effective in preventing demineralization. This result is in agreement with those observed in other investigations^23^^,^ 24 ^,^ 25 in which light-curing sealants and chemically activated ones provided the enamel with sufficient protection during the process of mineral loss. The authors believe that this reduction in protection by Pro Seal may be due to the curing process or composition of the product.

The investigation conducted by Shinaishin, Ghobashy and El-Bialy^25^ revealed that the group coated with Pro Seal obtained the lowest surface roughness values and total area exposed when compared to other groups. According to the authors, this suggests that the incorporation of load particles into the sealant appears to improve and increase the thin coat of product to be kept on the tooth surface during treatment, offering an adequate resistance to abrasion *in vivo*.

This study, similarly to the study by Farina et al,^23^ also suggests that Pro Seal appears to provide the enamel with protection against damage caused by changes in pH, preserving the organization of enamel prisms under normal conditions. This fact may justify the findings of the present study.

Based on the evidence provided by this research, it is possible to assert that fluoride sealant is a good adjuvant in clinical practice with the intention of preventing or significantly reducing the development of white spot lesions around orthodontic brackets, particularly in patients with impaired oral hygiene.

## CONCLUSION

By conducting this study, it could be concluded that Pro Seal sealant alone or combined with brushing and/or brushing and the use of mouthwash with fluoride was more effective in protecting enamel, in comparison to brushing alone.
